# The NAVETA obesity consensus tool: a multidimensional framework for clinical and patient-reported outcome assessment

**DOI:** 10.3389/fendo.2025.1731194

**Published:** 2026-01-08

**Authors:** Gabriel Mercadal-Orfila, Josep Lluch-Taltavull, Guillem Cuatrecasas, José María García, María Antonia Maestre-Fullana, Salvador Herrera-Pérez

**Affiliations:** 1 Pharmacy Department, Hospital Mateu Orfila, Mahon, Spain; 2 Department of Biochemistry and Molecular Biology, Universitat de les Illes Balears, Mallorca, Spain; 3 Open University of Catalonia (UOC) ‒ Group CPEN Endocrinology, Barcelona, Spain; 4 Group CPEN Endocrinology, Barcelona, Spain; 5 Unitat dʼObesitat, Hospital Quirón-Teknon, Barcelona, Spain; 6 Open University of Catalonia (UOC), Faculty of Health Sciences, Barcelona, Spain; 7 Department of General and Digestive Surgery, Hospital Mateu Orfila, Mahon, Spain; 8 Pharmacy Department, Hospital de Manacor, Manacor, Spain; 9 Faculty of Health Sciences, Universidad Internacional de Valencia, Valencia, Spain

**Keywords:** consensus framework, digital health, GLP-1 receptor agonists, health-related quality of life (HRQoL), obesity, patient-reported outcome measures (PROMs), patient-reported experience measures (PREMs), value-based healthcare (VBHC)

## Abstract

**Background:**

Obesity is a chronic, multifactorial, and relapsing disease that profoundly impairs physical, psychological, and social functioning, leading to substantial deterioration in health-related quality of life (HRQoL). Despite growing evidence on the clinical and organizational value of patient-reported outcome measures (PROMs) and patient-reported experience measures (PREMs), their systematic integration into obesity care—particularly within the Spanish healthcare system—remains limited.

**Objective:**

To develop the NAVETA Obesity Consensus Tool, a multidisciplinary, consensus-based framework that standardizes the multidimensional assessment of individuals living with obesity by integrating validated PROMs, PREMs, and clinical indicators within a digital, value-based healthcare (VBHC) model.

**Methods:**

Between October 2023 and January 2024, a multidisciplinary panel of experts in endocrinology, nutrition, bariatric surgery, hospital pharmacy, and psychology conducted a structured, iterative consensus process following modified Delphi principles. The development included a systematic literature review, expert scoring of candidate measures, and iterative feedback meetings. Feasibility, scientific validity, and patient relevance were evaluated in collaboration with a national patient association.

**Results:**

The final framework integrates two complementary domains (1): clinical and physiological variables organized into four categories—clinical, surgical, pharmacological, and nutritional–behavioral—and (2) patient-reported measures encompassing a standardized set of ten validated instruments in Spanish. The proposed PROMs/PREMs set captures key dimensions of obesity care, including quality of life (Moorehead–Ardelt QoLQ), mental health (PHQ-9), sleep quality (STOP-BANG, ISI), dietary adherence (MEDAS), pharmacological satisfaction and adherence (TSQM, MMAS-8), perceived discrimination (MSPD), stigma (SSI), and patient experience (IEXPAC).

**Conclusions:**

The NAVETA Obesity Consensus Tool represents the first consensus-based, patient-centered framework for standardized outcome assessment in obesity care in Spain. By combining validated PROMs, PREMs, and clinical variables within a digital platform, it provides a structured foundation for individualized follow-up, real-world data generation, and benchmarking across centers. This initiative supports the transition toward precision and value-based obesity management—particularly in the context of GLP-1 receptor agonist therapy—and lays the groundwork for a national outcome-based registry aligned with international standards.

## Introduction

Obesity is a chronic, multifactorial, and relapsing disease characterized by excessive adipose tissue accumulation, commonly defined by a body mass index (BMI) ≥30 kg/m² (1). It represents one of the most significant global health challenges due to its strong association with type 2 diabetes mellitus, dyslipidemia, hypertension, cardiovascular disease, certain cancers, and severe osteoarticular disorders—all contributing to substantial deterioration in health-related quality of life (HRQoL) (2, 3). Individuals with obesity frequently experience chronic pain, reduced mobility, fatigue, body-image dissatisfaction, and stigma, leading to lower physical and mental health scores compared with individuals of normal weight (4, 5). This multidimensional burden affects psychosocial functioning and everyday participation and reinforces the need for an integrated assessment that encompasses medical, surgical, pharmacological, and behavioral aspects (4, 6, 7).

Obesity also imposes a considerable economic and organizational burden on healthcare systems. In OECD countries, more than half of adults are overweight and one in four meet criteria for obesity, with related costs reaching up to 8% of total healthcare expenditure (8). Despite sustained public-health interventions, behavioral and structural determinants—such as physical inactivity, excessive caloric intake, and socioeconomic disparities—remain key contributors to this epidemic (5, 9, 10).

In recent years, glucagon-like peptide-1 receptor agonists (GLP-1 RAs) have transformed obesity pharmacotherapy. Originally developed for type 2 diabetes (11), agents such as liraglutide, semaglutide, and tirzepatide have shown unprecedented efficacy in inducing and sustaining weight loss through complementary central and peripheral mechanisms (12–14), with clinical trials reporting reductions of 10–15% for liraglutide and semaglutide and up to 25% for tirzepatide (15). Beyond weight loss, GLP-1 RAs improve metabolic control, reduce cardiometabolic risk, and enhance HRQoL (16), further underscoring the need for systematic monitoring of outcomes, adherence, and tolerability (17).

Simultaneously, healthcare systems are shifting toward value-based healthcare (VBHC) models (18–20), in which value is defined by patient-relevant outcomes relative to the resources required to achieve them (21). Patient-Reported Outcome Measures (PROMs) and Patient-Reported Experience Measures (PREMs) are central to this paradigm. PROMs capture patients’ own assessments of symptoms, functioning, and HRQoL (22, 23), whereas PREMs evaluate their experiences of care—communication, accessibility, continuity—which strongly influence engagement and adherence (24). Their routine use has been shown to strengthen shared decision-making and individualized care planning (25).

Despite robust evidence, routine integration of PROMs and PREMs in obesity care remains limited. Only a few countries (e.g., the UK, Denmark, the Netherlands) have established national PROM/PREM infrastructures, while implementation elsewhere is still fragmented due to challenges in standardization, data capture, and system-level adoption (26–28). In Spain, no widely adopted standard set for obesity currently exists, although initiatives such as BiblioPRO, local PROM protocols, and participation in OECD’s PaRIS project provide a favorable foundation (29–34).

To situate this initiative, the Naveta Obesity Scientific Committee is an independent, multidisciplinary working group established in 2023 within the broader NAVETA Value-Based Health initiative. Although not nationally appointed nor formally affiliated with a professional society, it functions as a self-organized expert body involving hospital pharmacy, endocrinology, nutrition, bariatric surgery, and digital-health teams across the Balearic Islands. Its remit is to define a structured set of clinical and patient-reported outcomes suited to digital follow-up pathways in the Spanish public health system.

In this context, we hypothesized that it is feasible to develop a structured digital tool based on validated PROMs/PREMs and current scientific evidence, centered on the needs and experiences of individuals living with obesity in Spain. We anticipate that such a model will enhance the quality of obesity care by enabling personalized monitoring, supporting multidisciplinary coordination, and integrating patient perspectives into therapeutic decision-making. The Naveta Obesity Consensus Tool is designed to support optimization and longitudinal follow-up—particularly in patients treated with GLP-1 RAs—while facilitating the transition toward VBHC. Accordingly, the main objective of this project is to develop and validate this consensus-based framework to serve as a comprehensive foundation for understanding patients’ needs, preferences, and values in the context of emerging pharmacological treatments for obesity in Spain.

## Methods

### Study design and objectives

The *Naveta Obesity Consensus Tool* was developed through a structured expert consensus aimed at establishing a standardized framework for the systematic collection of clinical, physiological, and patient-reported data in individuals with obesity, particularly in the context of treatment with GLP-1 RAs. The initiative sought to promote the integration of patient-reported outcomes and digital health solutions within clinical care pathways, thereby advancing toward a value-based healthcare (VBHC) model that balances outcomes with costs and prioritizes high-value interventions.

### Consensus framework and selection criteria

A structured, iterative consensus process—based on modified Delphi methodology—was conducted between October 2023 and January 2024. The procedure followed three coordinated phases (1): systematic literature review to identify candidate PROMs, PREMs, and clinical variables; (2) independent expert scoring and discussion; and (3) iterative consensus rounds to refine and finalize the standard set.

In the first round, the coordinators compiled an initial longlist of instruments across all domains. Alternatives were explicitly compared (e.g., Moorehead–Ardelt QoLQ vs. OWLQOL; PHQ-9 vs. HADS-D; IEXPAC vs. P3CEQ; STOP-BANG vs. Berlin Questionnaire). Each expert independently evaluated every candidate using five predefined criteria: (1) psychometric robustness (validity, reliability, sensitivity to change), (2) prior validation in obesity or metabolic disease, (3) availability of a validated or culturally adapted Spanish version, (4) feasibility for digital administration (no images, minimal skip logic, straightforward scoring), (5) patient burden, prioritizing shorter instruments to minimize fatigue when completing the full standard set.

After the first scoring round, an anonymized summary of quantitative scores and qualitative comments was distributed. The second round focused on structured discussion of discrepancies, particularly regarding instrument length, redundancy between PROMs measuring similar constructs, clinical interpretability, and user-friendliness in digital platforms. Measures scoring below the 75% agreement threshold were re-evaluated. Consensus was defined *a priori* as ≥80% agreement on inclusion, exclusion, or need for modification.

The third and final round consisted of a consensus meeting to resolve outstanding disagreements, ensuring adequate domain coverage while avoiding duplication. Unanimous agreement was ultimately achieved for the final standard set. This process ensured that all selected instruments met rigorous psychometric criteria, were feasible for digital implementation, and aligned with patient-centered usability considerations relevant to routine obesity care.

### Data domains and structure

The Naveta Obesity Consensus Tool integrates two complementary domains: Patient-reported measures – encompassing validated PROMs and PREMs to systematically capture patients’ perceptions of health status, quality of life, and care experience. Clinical and physiological measures – including anthropometric, metabolic, and treatment-related variables relevant to obesity and GLP-1 RAs therapy. Taken together, these domains form a multidimensional model for comprehensive, patient-centered assessment and monitoring.

### Expert committee composition

The Naveta Obesity Scientific Committee was formed specifically for this project and functions as a self-organized, multidisciplinary panel within the NAVETA Value-Based Healthcare program. Experts were selected through purposive sampling by the project coordinators (S. Herrera-Pérez and G. Mercadal-Orfila), based on predefined criteria: (a) recognized clinical and academic trajectory in their respective specialties; (b) extensive experience in the management of obesity across endocrinology, bariatric surgery, clinical nutrition, and hospital pharmacy—particularly in the pharmacological management of obesity with GLP-1 receptor agonists; and (c) methodological expertise in psychometrics and in the implementation of PROMs and PREMs in hospital settings. Although not formally endorsed by a national society, the Committee sought external validation from the national patient association AB Híspalis to ensure alignment with patient priorities and usability.

The Expert Committee comprised six professionals from diverse clinical and academic backgrounds, including endocrinology, nutrition, bariatric surgery, hospital pharmacy, and clinical psychology. Coordinators were Salvador Herrera-Pérez, PhD (Clinical Psychology, PROMs/PREMs and digital health) and Gabriel Mercadal, PhD (Hospital Pharmacy, clinical PROMs implementation). Experts included Josep Lluch Taltavull, RD (nutrition and QoL), Guillem Cuatrecasas, MD phD (endocrinology), José María García, MD (bariatric surgery), and M^a^ Antonia Maestre Fullana, PhD (hospital pharmacy).

The final version of the tool was reviewed by the Spanish Asociación Bariátrica Híspalis Nacional (AB Híspalis), the national patient association for individuals with obesity and bariatric surgery. The association conducted a document review and formally endorsed the tool, although no additional modifications were requested during this step.

### Ethical considerations and next steps

This expert consensus did not involve patient-level data and was therefore exempt from ethics approval. The resulting framework will serve as the basis for a digital pilot phase in selected centers to assess feasibility, acceptability, and longitudinal validity in real-world settings.

## Results

Following the expert consensus process, a structured set of variables was defined and organized into four major domains: Clinical, Surgical, Pharmacological, and Nutritional–Behavioral. These domains encompass the key dimensions necessary for a comprehensive and patient-centered assessment of obesity, integrating medical, functional, and psychosocial perspectives.

### Clinical, surgical, pharmacological, and nutritional–behavioral domains

#### Clinical domain

A comprehensive clinical assessment of patients with obesity requires consideration of both family and personal medical history, enabling the identification of predisposing risk factors and comorbid conditions essential for personalized treatment planning. The evaluation must address metabolic, cardiovascular, respiratory, musculoskeletal, and psychiatric comorbidities, all of which significantly influence health-related quality of life (HRQoL) and long-term prognosis.

Obesity is associated with a wide range of systemic complications. Among the most prevalent are arterial hypertension, which can lead to heart failure and chronic kidney disease; dyslipidemia, increasing the risk of atherosclerosis and cardiovascular events; and type 2 diabetes mellitus, in which obesity is a primary risk factor and weight control is key to glycemic management. In women, polycystic ovary syndrome (PCOS) frequently coexists with insulin resistance, aggravating metabolic and hormonal disturbances. Metabolic-associated fatty liver disease (MAFLD), formerly known as non-alcoholic fatty liver disease (NAFLD), is also common and may progress to cirrhosis if not addressed. Other frequent comorbidities include obstructive sleep apnea (OSA), osteoarthritis, gastroesophageal reflux disease (GERD), and malignancies such as endometrial, breast, and colon cancer. Psychiatric disorders, particularly depression and anxiety, are also prevalent and can negatively impact treatment adherence and health behaviors (1–7).

A detailed clinical assessment should include anthropometric and metabolic parameters, such as weight, height, body mass index (BMI), waist circumference, waist-to-height ratio (WHtR), and body fat percentage, the latter preferably measured using bioimpedance or dual-energy X-ray absorptiometry (DXA) (35). In addition, hepatic fibrosis should be screened using the FIB-4 index, a non-invasive marker combining age, AST, ALT, and platelet count. A FIB-4 <1.3indicates low risk of advanced fibrosis, whereas >2.67 suggests high risk and warrants further testing, such as elastography or biopsy (8). Finally, the Edmonton Obesity Staging System (EOSS) is recommended to stratify disease severity. This system evaluates not only BMI but also the presence and intensity of obesity-related comorbidities, categorizing patients into stages 0–4. EOSS provides clinically actionable information to guide treatment prioritization and tailor interventions to the individual’s health burden (9).

#### Surgical domain

The surgical domain includes variables related to prior bariatric procedures and associated surgical complications, providing insight into the long-term outcomes of surgical interventions and their interactions with pharmacological treatments. Relevant variables include the type and number of previous bariatric procedures, classification of complications using the Clavien–Dindo scale, and the occurrence of late postoperative complications such as gastroesophageal reflux, dumping syndrome, steatorrhea, dysphagia, or weight regain (10). Given the increasing use of GLP-1 RAs in post-bariatric patients, it is also recommended to monitor emergency visits related to gastrointestinal symptoms (e.g., epigastric pain, nausea, vomiting — potentially suggesting paralytic ileus —) or local adverse reactions (e.g., hematomas, infection, or bleeding at the injection site). These data are clinically relevant for evaluating treatment tolerance and optimizing long-term management (11–14).

#### Pharmacological domain

The GLP-1 RAs represent a major pharmacological innovation in the treatment of obesity and type 2 diabetes mellitus. These agents mimic the incretin hormone GLP-1, promoting satiety, delaying gastric emptying, and enhancing insulin secretion while suppressing glucagon release. They are associated with substantial improvements in both glycemic control and body weight (15–17).

Commonly used agents—liraglutide, semaglutide, and tirzepatide—differ in potency and pharmacokinetic profile but share a favorable risk–benefit ratio. Contraindications include a history of pancreatitis or medullary thyroid carcinoma. Special monitoring is advised in patients with cardiovascular disease, due to possible increases in heart rate, and in those with hepatic impairment, where risk of gallstones may rise secondary to rapid weight loss. GLP-1 RAs are generally well tolerated, though gastrointestinal adverse effects such as nausea (20–30%), vomiting (up to 24%), and diarrhea (15–22%) are common but transient (15, 18–20). Emergency visits for gastrointestinal or local injection-site complications should be recorded as indicators of treatment safety and tolerability. Tailoring therapy to individual risk profiles—particularly in older adults or patients with psychiatric comorbidities—enhances adherence and minimizes discontinuation.

#### Nutritional–behavioral domain

Behavioral re-education in dietary habits is a cornerstone of long-term obesity management. The primary goal is to modify maladaptive eating behaviors and establish sustainable, health-promoting habits. Key components include portion control, mindful eating, meal planning, and the adoption of nutrient-dense dietary patterns rich in vegetables, lean proteins, and whole grains (21). Evidence supports the efficacy of energy restriction (500–1000 kcal/day) and adherence to the Mediterranean diet in promoting weight loss and metabolic improvement. Structured, intensive behavioral interventions combined with regular follow-up have demonstrated significant and sustained effects on weight reduction and maintenance (22–24). Individualized dietary counseling, continuous reinforcement, and digital self-monitoring tools may further enhance patient engagement and adherence.

**Table 1** summarizes the consensus-derived set of clinical variables included in the NAVETA Obesity Consensus Tool. These variables were selected for their clinical relevance, feasibility, and applicability to the routine management of individuals with obesity, particularly those treated with GLP-1 receptor agonists. Organized into four complementary domains—clinical, surgical, pharmacological, and nutritional–behavioral—they provide a comprehensive framework for standardized, multidimensional assessment within value-based obesity care.

**Table 1:** Consensus clinical variables included in the NAVETA obesity consensus tool.

Domain	Variable	Description
Clinical History	Cardiovascular comorbidities	Includes hypertension, dyslipidemia, and type 2 diabetes mellitus, which are common in obesity and increase the risk of cardiovascular disease.
	Polycystic ovary syndrome (PCOS)	Common among women with obesity; associated with insulin resistance and metabolic complications.
	Hepatic steatosis	Accumulation of fat in the liver that may progress to cirrhosis.
	Obstructive sleep apnea (OSA)	Recurrent breathing interruptions during sleep that increase cardiovascular risk.
	Osteoarthritis	Degenerative joint disease exacerbated by excess body weight.
	Digestive diseases	Includes gastroesophageal reflux, pancreatitis, diverticulitis, and history of cholecystectomy.
	Psychiatric history	Includes depression and anxiety, which can negatively affect adherence to treatment.
Basic Parameters and EOSS	Weight, BMI, waist circumference, body fat percentage	Fundamental parameters for assessing obesity severity and its impact on health.
	Hepatic fibrosis (F) and FIB-4 index	Non-invasive assessment to estimate the risk of liver fibrosis in patients with obesity.
	Edmonton Obesity Staging System (EOSS)	Classification based on the physical, psychological, and functional impact of obesity, going beyond BMI.
Surgical Domain	Previous bariatric surgery procedures	Evaluation of the efficacy and complications of previous bariatric interventions.
	Surgical complications (Clavien–Dindo classification)	Categorization of postoperative complications according to their severity.
	Late surgical complications	Includes gastroesophageal reflux, dumping syndrome, weight regain, and other long-term complications after bariatric surgery.
	Emergency visits related to GLP-1 receptor agonist therapy	Monitoring of adverse effects associated with GLP-1 receptor agonists, such as gastrointestinal problems.
Pharmacological Treatment	GLP-1 receptor agonists	Evaluation of treatment type, dose, and duration, as well as tolerability and adherence.
	Adverse effects and tolerability	Recording of gastrointestinal and local adverse events (nausea, vomiting, diarrhea, injection-site reactions).
	Emergency visits due to treatment-related effects	Indicator of treatment safety and the need for therapeutic adjustment.
Nutritional–Behavioral Domain	Dietary behavior and education	Assessment of eating habits, portion control, and mindful eating practices.
	Nutritional pattern	Evaluation of adherence to healthy dietary models, such as the Mediterranean diet.
	Caloric intake and energy restriction	Recommendation of an energy deficit of 500–1000 kcal/day for weight reduction.
	Adherence and follow-up	Continuous monitoring and behavioral reinforcement to ensure long-term adherence to dietary and lifestyle modifications.

In addition to the core domains previously described, several additional aspects should be monitored throughout the follow-up of patients with obesity receiving GLP-1 receptor agonist therapy. These include pharmacological variables, such as the type and tolerability of GLP-1 receptor agonists, given their potential gastrointestinal adverse effects, as well as key elements of nutritional and behavioral management. The latter encompasses portion control, promoting appropriate serving sizes to regulate food intake; meal planning and preparation, encouraging healthier food choices and structured dietary habits; and nutrient-dense food selection, prioritizing vegetables, lean proteins, and whole grains. Together, these elements provide a comprehensive framework for optimizing metabolic outcomes, enhancing treatment adherence, and supporting long-term weight maintenance (16, 17, 25, 26).

### Health-related quality of life in patients with obesity

Health-related quality of life (HRQoL) has become a critical dimension in the comprehensive assessment of obesity, capturing the lived experience and functional impact of the disease beyond traditional biomedical indicators. Obesity adversely affects physical, psychological, emotional, and social functioning, often leading to pain, fatigue, reduced mobility, depressive symptoms, and impaired self-esteem. These effects are not only driven by metabolic and mechanical complications but also by pervasive stigma and discrimination, which further erode mental well-being and social participation (27–30). Consequently, individuals with obesity frequently report lower HRQoL scores across all domains compared with normal-weight populations (31). In this context, PROMs provide a standardized, patient-centered approach to quantify these multidimensional burdens, enabling clinicians to monitor progress, guide individualized interventions, and evaluate the broader impact of therapeutic strategies such as GLP-1 receptor agonists. The following sections summarize the main HRQoL domains most affected in individuals living with obesity.

#### Physical health

Obesity is strongly associated with numerous chronic diseases, including ischemic heart disease, heart failure, hypertension, type 2 diabetes mellitus, renal impairment, obstructive sleep apnea, musculoskeletal disorders, hyperuricemia, and certain cancers. These comorbidities contribute to pain, reduced mobility, fatigue, and shortened life expectancy. Obesity has also been linked to increased risk of stroke and multiple cancers, leading to substantial physical impairment and reduced daily functioning (32).

#### Mental health

The psychological impact of obesity is considerable. Stigma, discrimination, and negative self-perception commonly result in low self-esteem, depression, and anxiety. Studies have shown that individuals with obesity are more likely to experience psychological distress compared to those with normal weight. Regarding pharmacological management, GLP-1 RAs —widely prescribed for obesity and type 2 diabetes— have been investigated for potential associations with suicidal ideation. However, a large-scale cohort study found no increased risk of suicide among GLP-1 RA users compared with SGLT2 inhibitor users, and the U.S. FDA has not identified conclusive evidence of causality (33). Nevertheless, clinicians should monitor patients for emerging or worsening depressive symptoms, suicidal thoughts, or behavioral changes (34).

#### Emotional well-being

Obesity can hinder participation in social and recreational activities due to fatigue, pain, or self-consciousness. Social withdrawal may lead to reduced social reinforcement, exacerbating emotional distress. Studies published in the Journal of Health Psychology report that individuals with obesity experience lower social support and higher social isolation compared with non-obese counterparts, which may perpetuate a negative emotional cycle affecting HRQoL (36).

#### Interpersonal relationships

Weight-related stigma can affect personal and professional relationships (37). Individuals with obesity frequently report discrimination in social settings, the workplace, and even within family environments. This stigmatization may contribute to feelings of loneliness and reduced social integration. Research has shown that individuals with obesity experience significantly higher levels of perceived discrimination and prejudice compared with those of normal weight (38).

#### Sleep quality

Poor sleep quality further compounds metabolic dysregulation and impairs overall HRQoL (39). Obesity is a major risk factor for sleep disorders, particularly obstructive sleep apnea (OSA) and hypoventilation syndrome. Studies show that individuals with obesity have a significantly higher prevalence of sleep disturbances, including OSA, compared to non-obese individuals. For example, research found that around 19.6% of obese older adults suffer from sleep disorders, markedly higher than in normal weight or overweight peers, demonstrating a dose-response relationship between BMI and sleep disturbances (40).

In summary, obesity negatively affects HRQoL across physical, psychological, emotional, social, and economic dimensions. A value-based, patient-centered approach—supported by PROMs and PREMs—enables the systematic assessment of these domains in clinical practice.

### Proposed standard set

**Figure 1:**
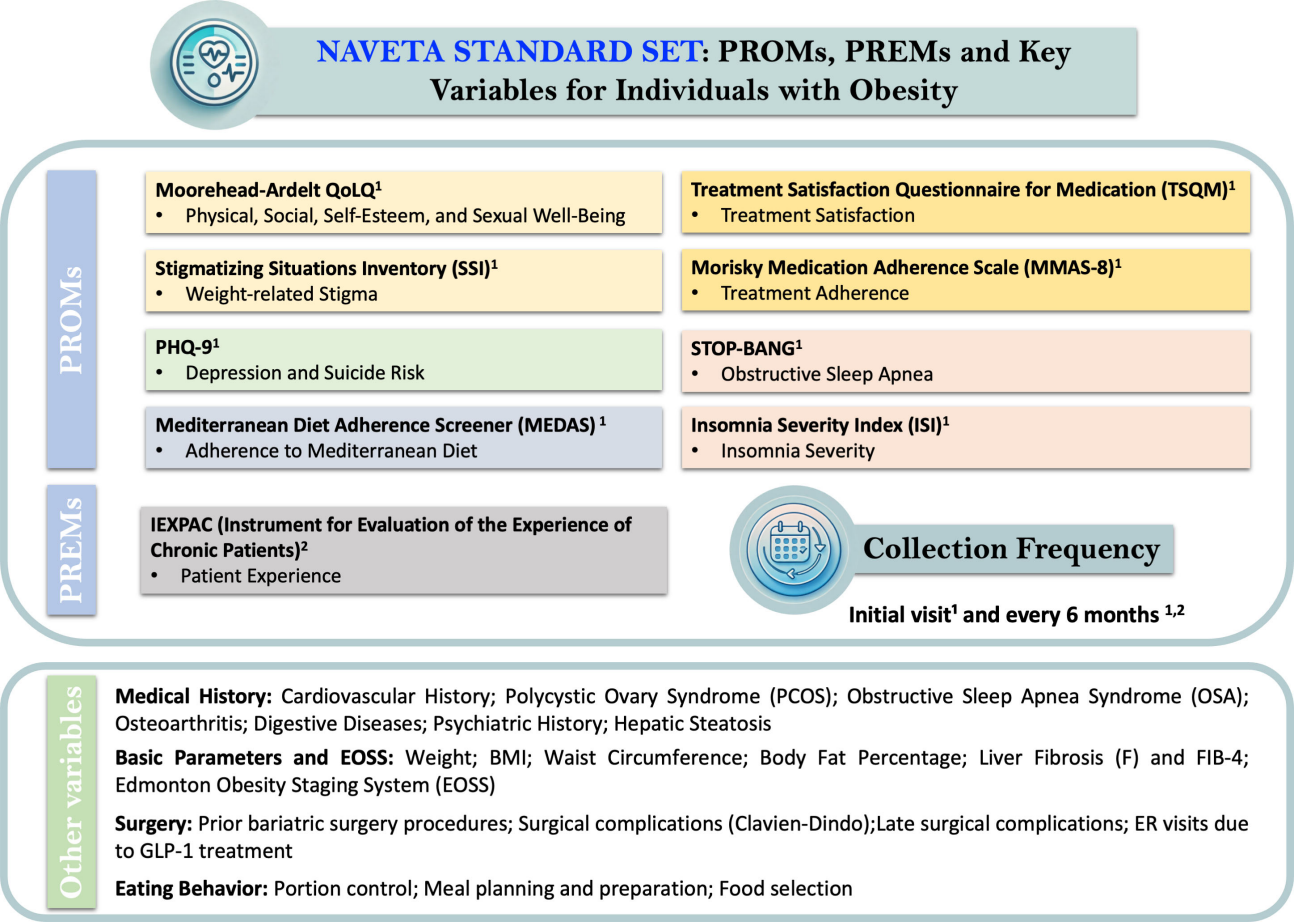
NAVETA standard set: PROMs, PREMs and key variables for individuals with obesity. Overview of the *NAVETA Obesity Consensus Standard Set*, which integrates validated Patient-Reported Outcome Measures (PROMs) and Patient-Reported Experience Measures (PREMs), alongside key clinical and behavioral variables for comprehensive assessment in individuals with obesity. The standard set includes ten validated instruments covering domains such as quality of life (Moorehead–Ardelt QoLQ), weight-related stigma (SSI), depression (PHQ-9), sleep disorders (STOP-BANG, ISI), dietary adherence (MEDAS), treatment satisfaction and adherence (TSQM, MMAS-8), and patient experience (IEXPAC). Complementary variables include clinical history, anthropometric parameters, liver fibrosis assessment, surgical history, and eating behavior. PROMs and PREMs are recommended for collection at the initial visit and every six months, supporting continuous, value-based, and patient-centered care monitoring.

The broader body of theoretical and empirical literature consistently supports the feasibility and clinical relevance of integrating patient-reported outcomes into routine obesity management. On this basis, the NAVETA Obesity Expert Committee has developed a consensus-derived standard set of PROMs and PREMs, tailored to assess health-related quality of life and care experience in individuals living with obesity. Based on the consensus process, a structured standard set of PROMs and PREMs was defined to comprehensively assess HRQoL in individuals with obesity (**Figure 1**, **Table 2**). The selection prioritized validated instruments with available Spanish versions, appropriate psychometric properties, and feasibility for clinical use.

**Table 2:** Recommended standard set.

Questionnaire	Dimensions assessed	Number of items
Moorehead–Ardelt QoLQ	- Physical Dimension - Social Dimension - Self-Esteem - Sexual Dimension	6
Stigmatizing Situations Inventory (SSI)	- Weight-Related Stigma	10
PHQ-9	- Psychological Dimension (Depression and Suicide Risk)	9
Stop-Bang	- Sleep Dimension (Obstructive Sleep Apnea)	8
Insomnia Severity Index (ISI)	- Sleep Dimension (Sleep Quality and Insomnia Severity)	7
Mediterranean Diet Adherence Screener (MEDAS)	- Nutritional Dimension – Adherence to the Mediterranean Diet	14
Treatment Satisfaction Questionnaire for Medication (TSQM)	- Pharmacological Treatment (Effectiveness, Convenience, Side Effects, and Global Satisfaction)	14
Morisky Medication Adherence Scale (MMAS-8)	- Treatment Adherence	8
Multidimensional Scale of Perceived Discrimination (MSPD)	- Perceived Discrimination	20
IEXPAC (Instrument for the Evaluation of the Experience of Chronic Patients)	- Patient Experience	15

Summary of the standardized set of Patient-Reported Outcome Measures (PROMs) and Patient-Reported Experience Measures (PREMs) proposed by the *NAVETA Obesity Expert Committee*.

During the initial instrument-selection phase of the consensus process, several obesity-specific PROMs were screened, including OWLQOL (Obesity and Weight-Loss Quality of Life Questionnaire) and other established measures. Although these instruments exhibit solid psychometric properties in the literature, they were not taken forward due to limitations identified during the early appraisal stage—namely their greater length, the limited availability of validated Spanish versions, and their lower feasibility for digital administration compared with the instruments retained in the proposed standard set. The committee also considered whether a shorter core set might be sufficient; however, it became evident in the early rounds that capturing the full breadth of psychosocial, behavioral, sleep-related, and treatment-related domains required a multidimensional structure. Feasibility concerns were addressed by prioritizing brief, validated instruments during the screening phase, and by noting that future pilot testing within NAVETA will evaluate patient burden, completion rates, and the potential need for further refinement.

*The Moorehead–Ardelt Quality of Life Questionnaire (QoLQ; 6 items)* evaluates physical, social, self-esteem, and sexual domains, offering a concise measure of obesity-specific quality of life frequently used in bariatric populations (41). The M-A QoLQ has demonstrated robust validity and reliability for assessing quality of life in patients undergoing bariatric surgery and, more broadly, in individuals with obesity. The Spanish version shows strong internal consistency, with Cronbach’s alpha values ranging from 0.817 to 0.885, levels considered high and comparable to those reported in other European language adaptations. Construct validity has been supported through significant correlations with established HRQoL instruments such as the SF-36, and the questionnaire has shown adequate sensitivity to clinical change and reproducibility across time, confirming its suitability for longitudinal monitoring in obesity populations (42).

The Stigmatizing Situations Inventory (SSI; 10 items) and the Multidimensional Scale of Perceived Discrimination (MSPD; 20 items) capture perceived weight-related stigma and discrimination, dimensions often underassessed yet critical for psychosocial outcomes. The SSI shows good psychometric performance, including adequate internal consistency (Cronbach’s alpha >0.80 in both original and Spanish-adapted versions) and expected convergent associations with constructs such as BMI, self-esteem, and eating disorder symptomatology, supporting its sensitivity to psychosocial correlates of weight stigma (43, 44). The brief version (SSI-B) preserves the content validity and reliability of the full instrument, facilitating use in settings requiring shorter assessments. The MSPD has been applied in Spanish-speaking populations and demonstrates adequate internal consistency across its core dimensions, together with construct validity consistent with theoretical models of perceived discrimination (45, 46).

To assess mental health, the *Patient Health Questionnaire (PHQ-9; 9 items)* measures depressive symptoms and suicide risk, while sleep-related quality is evaluated using the STOP-BANG (8 items), a screening tool for obstructive sleep apnea, and the Insomnia Severity Index (ISI; 7 items), which quantifies sleep quality and insomnia severity (47, 48). The PHQ-9 shows robust psychometric performance in Spanish-speaking populations, including studies conducted in Spain and Latin America, confirming its reliability, validity, and suitability for assessing depressive symptoms in diverse clinical and community settings (49). The Spanish adaptation demonstrates good internal consistency, with Cronbach’s alpha values typically ranging between 0.80 and 0.86, and exhibits expected convergent validity patterns with established HRQoL and depression severity measures, supporting its capacity to capture the multidimensional impact of depressive symptoms (49, 50). Collectively, these findings highlight the PHQ-9 as a reliable, valid, and sensitive instrument for detecting and monitoring depression across clinical practice and research contexts (50).

*The STOP-BANG questionnaire* is a widely used screening tool for assessing the risk of obstructive sleep apnea (OSA), consisting of eight key items addressing major clinical risk factors. The acronym STOP-BANG represents snoring, tiredness, observed apnea, pressure (hypertension), body mass index (BMI >35 kg/m²), Age (>50 years), Neck circumference (>40 cm), and gender (male). Each affirmative response scores one point, yielding a total score between 0 and 8, with scores of 0–2 indicating low risk, 3–4 intermediate risk, and 5–8 high risk of moderate-to-severe OSA. The Spanish version has undergone formal cross-cultural adaptation and shows adequate internal consistency, with Cronbach’s alpha values above 0.76 in validation studies (51). In terms of psychometric performance, the STOP-BANG demonstrates good screening accuracy for identifying individuals at risk of moderate-to-severe OSA, and its brevity and ease of administration support its suitability for routine clinical use. Although false-positive rates may be higher in certain clinical subgroups, the questionnaire remains one of the most practical and widely validated tools for OSA risk assessment in both clinical and research settings (52).Nutritional habits are assessed through the *Mediterranean Diet Adherence Screener* (MEDAS; 14 items), a validated tool developed within the PREDIMED study and widely used in Spain to evaluate adherence to the Mediterranean dietary pattern (53). This brief questionnaire assesses the intake of core Mediterranean diet components, including olive oil as the primary fat source, vegetables, fruits, legumes, fish and seafood, nuts, and moderate wine consumption, while discouraging red and processed meats. The MEDAS demonstrates strong psychometric performance in Spanish populations, with high test–retest reliability (ICC = 0.876; 95% CI: 0.831–0.909) and good criterion validity, evidenced by significant correlations with the MedQ-Sus comparative score. It also shows discriminant validity, effectively differentiating between adherence and non-adherence, with an optimal cut-off of 7.5, sensitivity of 0.81, specificity of 0.57, and an AUC of 0.743 (54–57).

Pharmacological treatment satisfaction and adherence are captured with the Treatment *Satisfaction Questionnaire for Medication (TSQM; 14 items)*—which evaluates effectiveness, convenience, side effects, and overall satisfaction—and the Morisky *Medication Adherence Scale (MMAS-8; 8 items)*, respectively (58–60). Both instruments have validated Spanish versions, robust psychometric support, and broad acceptance for use in Spanish-speaking clinical and research settings (61, 62).The TSQM demonstrates high internal consistency across its domains (Cronbach’s alpha typically >0.80) and strong construct validity, supporting its ability to capture multiple dimensions of treatment satisfaction. It also shows sensitivity to clinical change, making it a useful instrument for monitoring patients’ perceptions of pharmacological therapy (63). The MMAS-8 likewise exhibits adequate internal consistency (alpha values generally above 0.80), acceptable test–retest reliability, and established construct validity for assessing medication-taking behavior, reinforcing its suitability for evaluating adherence in both clinical practice and research contexts (60).

Finally, the IEXPAC questionnaire (15 items), developed and validated in Spain, assesses patients’ experiences with chronic disease management and the healthcare system, aligning the evaluation with the principles of Value-Based Healthcare (VBHC) (64).

Together, these ten instruments provide a robust and multidimensional framework for evaluating outcomes and patient experiences in obesity care, facilitating comparability across clinical settings and supporting the integration of PROMs and PREMs into digital follow-up systems.

## Discussion

The present work describes the development of the NAVETA Obesity Consensus Tool, a structured framework designed to standardize the assessment of patient-reported outcomes and experiences in individuals living with obesity. The primary aim of this initiative was to establish a consensus-based standard set of PROMs and PREMs that complements clinical and behavioral data, thereby supporting patient-centered and value-based care models. Building on a pragmatic, multi-stakeholder process, the tool is intended for both point-of-care use and service evaluation, enabling clinicians and managers to monitor what matters most to patients while ensuring comparability across sites and care pathways.

The consensus process, led by a multidisciplinary expert committee, resulted in the identification of ten validated and psychometrically robust instruments that collectively capture the broad impact of obesity—spanning physical, psychological, social, nutritional, pharmacological, and experiential domains. The inclusion of both PROMs (e.g., Moorehead–Ardelt QoLQ, PHQ-9, MEDAS, TSQM, MMAS-8) and PREMs (IEXPAC) ensures that not only HRQoL but also patient experience and engagement are systematically monitored. This multidomain approach aligns with international initiatives such as the ICHOM Obesity Standard Set and the OECD’s PaRIS project, emphasizing the importance of patient-reported data for benchmarking quality and improving healthcare system performance. Such alignment facilitates international comparability while preserving local feasibility through the use of Spanish-validated versions and digitally supported administration.

Several multidimensional frameworks for obesity assessment have been proposed previously, including the core PROM set for obesity treatment research by (65), the set of PROMs recommended for the Metabolic and Bariatric Surgery Accreditation Quality Improvement Program (66), and the international core outcome set developed through a modified Delphi process by (67). Our framework aligns with these initiatives in emphasizing patient-centered outcomes but differs by focusing on the Spanish healthcare context, integrating PROMs and PREMs together with clinical variables, and applying selection criteria based on Spanish-language availability, digital feasibility, and patient burden. While our approach prioritizes local applicability within a digital value-based care model, the growing number of international efforts underscores the desirability of future harmonization to support comparability, benchmarking, and cross-country research.

Compared with existing efforts, the NAVETA initiative makes two distinctive contributions (26–28). First, it provides a harmonized selection of validated instruments already available in Spanish, addressing a major barrier to implementation in Spain and other Spanish-speaking countries. Second, integrating PROMs and PREMs within a digital health framework enables continuous monitoring and facilitates real-world data collection in support of value-based healthcare (98,99). Embedding the set in electronic workflows may reduce missing data, generate automated reminders, and produce dashboards combining clinical indicators with PROMs trajectories, supporting shared decision-making and multidisciplinary coordination.

Beyond its methodological rigor, the NAVETA framework responds to an unmet clinical need: aligning obesity management with outcome-based principles that emphasize patient relevance and multidimensional benefit. In traditional models, success has been defined largely by weight reduction or biochemical improvement; however, these metrics fail to capture the patient’s lived experience, determinants of adherence, and functional recovery (68). By incorporating standardized PROMs and PREMs, NAVETA reframes outcome evaluation around parameters that genuinely reflect value for patients—improvements in energy, mood, self-perception, and participation in social and occupational activities. This shift is particularly relevant in the era of GLP-1 receptor agonists (GLP-1 RAs), where substantial weight loss can coexist with variable satisfaction, adherence, or psychological adjustment to body changes. A combined assessment integrating clinical endpoints and patient-reported outcomes thus offers a more comprehensive and precise measure of therapeutic success.

The framework also fills a methodological gap within the Spanish healthcare landscape. Although PROMs and PREMs have gained traction in oncology, cardiology, and rheumatology, their integration into obesity management remains scarce and heterogeneous (69, 70). NAVETA serves as a catalyst for creating interoperable registries linking patient perspectives with clinical outcomes and cost indicators, contributing to more transparent and equitable healthcare resource allocation. Its interoperable design promotes harmonization across decentralized regional systems without compromising local adaptability.

From an implementation standpoint, several challenges must be addressed. Digital literacy, for both patients and healthcare professionals, is a key determinant of success (71). Completing multiple questionnaires may generate fatigue, particularly among older or socioeconomically disadvantaged populations. Nevertheless, prior studies indicate that brief, well-designed PROMs administered via intuitive mobile interfaces can achieve high completion rates and reliability (72). Future iterations of the NAVETA platform could employ adaptive testing or artificial intelligence algorithms to tailor item presentation dynamically, minimizing redundancy while maintaining psychometric rigor (73).

In addition, although the expert committee prioritized brief instruments during the selection process, the simultaneous administration of ten PROMs/PREMs inevitably raises concerns about patient burden. For this reason, the panel discussed the possibility of defining a shorter core set, an aspect that will be revisited during the implementation phase. Future NAVETA pilot testing will evaluate burden, completion rates, and user acceptability, and will explore strategies to reduce perceived load—such as distributing questionnaires across multiple short timepoints or using adaptive scheduling to deliver measures in smaller, clinically meaningful clusters without compromising the integrity of longitudinal assessment. Finally, operational aspects such as baseline versus follow-up workflows, alert mechanisms, handling of missing responses, or expected completion time were not part of the present consensus process and will be defined in upcoming NAVETA pilot and implementation studies.

Beyond feasibility considerations, another limitation concerns the scope of the consensus process itself. Although the expert panel was multidisciplinary and included patient representation, the sample size was limited and perspectives from primary care and public health practitioners were underrepresented. The next phase should therefore broaden stakeholder involvement—particularly from general practitioners, nurses, and health-system decision-makers—to ensure that the tool remains clinically meaningful and operationally feasible across diverse care settings. Additionally, because the committee operated as a small collaborative group, full anonymity between experts was not implemented during the evaluation rounds. While this departs from the classical Delphi model, it is consistent with commonly applied modified Delphi approaches in clinical consensus development and did not compromise the transparency or independence of the decision-making process. Despite these limitations, the NAVETA Obesity Consensus Tool presents several strengths. It is grounded in validated measures supported by psychometric and clinical evidence and aligned with international standards. Its integration of physical, behavioral, and experiential data reflects an evolution from a purely biomedical toward a biopsychosocial and outcome-based understanding of obesity. The tool’s modular structure enhances flexibility, allowing adaptation to different clinical contexts—from pharmacological therapy monitoring to postoperative follow-up in bariatric surgery. Additionally, by including PREMs such as IEXPAC, the model explicitly recognizes the importance of patient–professional interaction, organizational continuity, and empowerment as determinants of long-term success.

In practice, systematic use of NAVETA Obesity Consensus Tool could inform clinical decision-making, enable benchmarking through inter-center comparisons, and generate high-quality, real-world datasets for predictive analytics, including models of adherence, relapse, or remission trajectories. Implementing PROMs and PREMs within digital clinical environments also has ethical and social implications. Capturing patient perspectives systematically helps reduce stigma by demonstrating that subjective experiences are valued in clinical encounters. Transparent communication of aggregated PROMs data can foster accountability and trust within healthcare institutions, consistent with the broader European agenda for patient empowerment and personalized medicine.

As the NAVETA Obesity Consensus Tool begins to be implemented and real-world data accumulate, an important next step will be to examine how each included measure performs psychometrically in individuals with obesity and how the different PROMs and PREMs relate to one another in routine clinical settings. This will help determine the stability, responsiveness, and inter-domain coherence of the standard set and will inform potential refinements. In particular, analyzing completion patterns, redundancy across domains, and item-level behavior may support the development of a shorter or adaptive version of the battery, improving feasibility while maintaining conceptual coverage. Incorporating these evaluations into future NAVETA studies will position the tool as a dynamic, evolving framework grounded in empirical evidence rather than a static endpoint.

In summary, the NAVETA Obesity Consensus Tool represents a pragmatic and forward-looking contribution to obesity care in Spain. It bridges the gap between evidence-based medicine and patient-centered practice by translating the principles of VBHC into measurable, actionable indicators. The next steps—digital piloting, longitudinal validation, and incorporation into national outcome registries—will determine its scalability and real-world impact. Nonetheless, the consensus achieved provides a solid foundation for transforming obesity management from a fragmented, weight-centric model into one guided by multidimensional, patient-valued outcomes.

## Conclusions

In conclusion, the NAVETA Obesity Consensus Tool provides the first consensus-based framework in Spain for the standardized and patient-centered assessment of individuals living with obesity, particularly in the context of pharmacological treatment with GLP-1 receptor agonists. By combining validated PROMs, PREMs, and key clinical variables, this model establishes a structured foundation for monitoring treatment outcomes and optimizing therapeutic decisions. Furthermore, it lays the groundwork for the development of a national outcome-based registry and supports the transition toward value-based healthcare in obesity management. Future steps will focus on piloting digital implementation, validating longitudinal responsiveness, and promoting adoption across clinical networks to enhance long-term follow-up and real-world data generation. In the long term, the NAVETA framework may contribute to harmonizing clinical practice, strengthening research on patient-reported outcomes, and informing policy decisions that foster equitable, high-value obesity care across the Spanish healthcare system.

## Data Availability

The original contributions presented in the study are included in the article/supplementary material. Further inquiries can be directed to the corresponding author.
